# Surface Modification of ETFE Membrane and PTFE Membrane by Atmospheric DBD Plasma

**DOI:** 10.3390/membranes12050510

**Published:** 2022-05-10

**Authors:** Zuohui Ji, Yue Zhao, Min Zhang, Xiaopeng Li, Heguo Li

**Affiliations:** State Key Laboratory of NBC Protection for Civilian, Institute of Chemical Defense, Beijing 100191, China; zuohui1210@163.com (Z.J.); sa11226532@mail.ustc.edu.cn (Y.Z.); minjohng@126.com (M.Z.); lxpbuct@163.com (X.L.)

**Keywords:** DBD plasma, surface modification, hydrophilicity, adhesion strength, fluorine resin membrane

## Abstract

Fluorine resin membranes with excellent chemical resistance have great potential for the application of high-performance chemical protective clothing. However, it is difficult to integrate fluorine resins into other materials such as fabrics due to their lower surface energy and poor bondability, making the fabrication of composite fabrics and the further seal splicing challenging. In this study, atmospheric pressure dielectric barrier discharge (DBD) plasma in helium (He) and helium/acrylic acid (He/AA) mixture atmospheres were used to modify two kinds of fluorine resins, ethylene tetrafluoroethylene (ETFE) and polytetrafluoroethylene (PTFE) membrane. The surface chemical properties, physical morphology, hydrophilicity and adhesion strength of the fluororesin membranes before and after plasma treatments were systematically analyzed. The results showed that the plasma treatment can modify the membrane surface at the nanoscale level without damaging the main body of the membrane. The hydrophilicity of the plasma-treated membrane was improved with the water contact angle decreasing from 95.83° to 49.9° for the ETFE membrane and from 109.9° to 67.8° for the PTFE membrane, respectively. The He plasma creates active sites on the membrane surface as well as etching the membrane surface, increasing the surface roughness. The He/AA plasma treatment introduces two types of polyacrylic acid (PAA)—deposited polyacrylic acid (d-PAA) and grafted polyacrylic acid (g-PAA)—on the membrane surface. Even after ultrasonic washing with acetone, g-PAA still existed stably and, as a result, improved the polarity and adhesion strength of fluororesin membranes. This work provides useful insights into the modification mechanism of DBD plasma on fluorine resins, with implications for developing effective strategies of integrating fluorine resin membrane to chemical protective clothing fabrics.

## 1. Introduction

With the development of the military and chemical industries, the threat of new chemical warfare agents and hazardous chemicals in industrial production have put forward higher protective requirements for chemical protective clothing (CPC). CPC can provide effective protection for the wearer in chemical warfare, industrial chemical production and hazardous-chemical disposal, preventing toxic and harmful agents from penetrating into the human body in the form of liquids, vapors and aerosols, thus protecting the health and lives of personnel [1,2,3,4]. CPC should have the ability to resist the penetration of toxic and hazardous chemicals. The traditional CPC made of rubber material, according to the principle of similar solubility, struggles to protect against some chemicals such as acetone, ethyl acetate and methylene chloride. Fluororesin with excellent chemical permeation resistance and chemical stability for its strong electronegativity and low polarizability can be used for an ideal high-barrier protective material, with great potential for high-level CPC fabrics. However, fluororesin is considerably challenging for chemical modification due to its low surface energy, non-polarity and poor bondability, limiting its application in protective clothing and other bonding fields.

In order to improve the surface energy and polarity of fluororesin, it is necessary to modify its surface. Common traditional surface-modification methods such as sodium naphthalene chemical treatment, the high-temperature fusion method, the irradiation grafting method, etc., have the advantages of simple operation and being universal and efficient. However, these treatment approaches may readily damage the structure of fluorine resin and cause environmental pollution. As a dry treatment method, plasma treatment is widely used in the field of the surface modification of polymer materials, which can introduce a variety of active functional groups on the surface of treated samples in a short time, with the advantages of low environmental pollution and a good modification effect. Current research on plasma modification of polymer-membrane surfaces is divided into low-pressure and atmospheric-pressure plasma treatment [5,6,7]. Low-pressure plasma treatment needs to be carried out in a certain vacuum environment, requiring corresponding vacuum equipment, which is demanding for special equipment and not suitable for large-scale processing. Atmospheric-pressure plasma can be carried out at atmospheric pressure, simplifying the modification process, reducing the production and operating costs, and facilitating the development of large-scale industrial modification production [8,9,10].

In this research, non-perfluorinated ethylene tetrafluoroethylene (ETFE) and perfluorinated polytetrafluoroethylene (PTFE) membranes were modified by atmospheric-pressure dielectric-barrier-discharge (DBD) plasma. Since the C–F bond energy is greater than the C–H bond energy, the perfluorinated PTFE needs more energy to break the C–F bond than the non-perfluorinated ETFE under the same conditions. In order to achieve a uniform and stable discharge state of DBD plasma at atmospheric pressure and enhance the stability of the surface-modification effect, two atmospheres, helium (He) and helium/acrylic acid (He/AA) mixture, were used as the treatment atmosphere [11,12], and the treatment effect of the two atmospheres was compared and analyzed. Additionally, the plasma-treated samples were ultrasonically washed with acetone to test the stability of the modified membrane surface. Attenuated total reflectance Fourier transform infrared spectroscopy (ATR-FTIR), X-ray photoelectron spectroscopy (XPS), atomic force microscopy (AFM), and scanning electron microscopy (SEM) were used to characterize the functional groups, elemental content, and morphology of the membrane surfaces before and after DBD plasma treatment. The mechanism of surface modification was unraveled based on the characterization results. The water contact angle test and T-peel test were conducted to analyze the effects of plasma modifications on membrane hydrophilicity and adhesion strength. The results showed that the bondabilities of ETFE and PTFE membranes were significantly improved using plasma treatments, which provides the great possibility of integrating fluororesin membranes with other materials to fabricate chemical protective clothing.

## 2. Materials and Methods

### 2.1. Materials

ETFE membrane (EFC-0055M) and PTFE membrane (PTFE-050) from Daikin Industries (Shanghai, China) with a thickness of about 50 μm were used in this research. Acrylic acid (CH_2_CHCOOH, AR), acetone (CH_3_COCH_3_, AR), and sodium hydroxide (NaOH, AR) were purchased from Sinopharm Chemical Reagent Co., Ltd (Shanghai, China). All chemicals were used as received without further purification. Helium (>99.999%) was obtained from Beijing Haipu Gas Co., Ltd (Beijing, China).

### 2.2. Plasma Treatment

**Figure 1:**
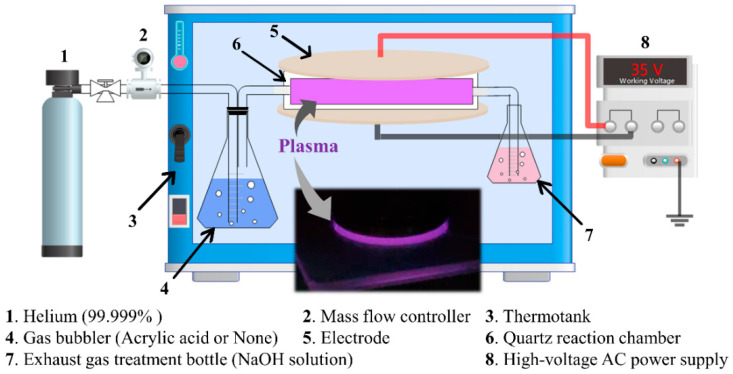
Atmospheric-pressure DBD plasma experimental device.

The ETFE and PTFE membranes were ultrasonically washed in acetone for 20 min to remove contaminants from the membrane surface, and then dried in use. The plasma treatment experimental device has two parts: the gas-generation part and the DBD-discharge part, as shown in Figure 1. In the gas-generation part, the gas flow rate is controlled by a mass flow controller to 200 mL/min. When the gas bubbler is not filled with any solvent, the plasma treatment atmosphere is pure helium. When the gas bubbler contains 30 mL of acrylic acid, the plasma treatment atmosphere is a He/AA mixture. In the DBD discharge section, the output of the high-voltage AC power supply (Equipment Model: CTP-2000K, Nanjing Suman Plasma Technology Co., Ltd., Nanjing, China) is connected to the two electrodes. The quartz reaction chamber sandwich between the two electrodes can be used as a blocking medium to avoid the generation of arcs and to separate the treatment atmosphere from the air. Membrane samples are placed in the quartz reaction chamber for plasma treatment. The acrylic acid in the exhaust gas is absorbed by the exhaust gas treatment bottle filled with sodium hydroxide solution. In order to ensure the stability of the gas concentration during the plasma treatment of fluororesin membrane experiments and that the plasma discharge process is not affected by temperature changes, part of the experimental device was placed in a constant thermotank at 25 °C.

Atmospheric-pressure DBD plasma surface modification was carried out under a He and He/AA mixed atmosphere at a controlled gas flow rate of 200 mL/min, respectively. To investigate the stability of the modification effect on the surface of membranes after He/AA plasma treatment, the partially modified membranes were ultrasonically washed in acetone for 20 min before characterization. The specific sample numbers discussed in this work and corresponding experimental conditions are shown in Table 1.

### 2.3. Characterization

The chemistry of the membrane was characterized via Fourier transform infrared spectroscopy (FTIR, Frontier IR, PerkinElmer, Waltham, MA, USA) using the attenuated total reflectance (ATR) mode. An atomic force microscope with a vision–infrared–Raman coupled system (AFM, VistaScope, Molecular Vista Inc., San Jose, CA, USA) based on the nano-infrared technology of photoinduced force microscopy (IR PiFM) was used to detect the nano-level infrared spectral characteristics of membrane surfaces before and after modification. The elemental composition of the membrane surface was examined via X-ray photoelectron spectroscopy (XPS, Thermo Scientific K-Alpha, Waltham, MA, USA). In order to explore the depth of plasma modification, an Ar gas cluster ion beam was used to etch the surface for 0, 0.5, 1.0, and 1.5 min, respectively, and then the elemental composition of the membrane surface was characterized by XPS (PHI5000 Versaprobe III, Ulvac-Phi Inc., Kanagawa, Japan). The membrane surface was coated with gold for 30 s and then observed by a scanning electron microscope (SEM, Gemini 300, Oberkochen, Germany). Atomic force microscopy (AFM, Dimension Icon, Bruker, Santa Barbara, CA, USA) was used to analyze the surface morphology of the membrane. A contact angle tensiometer (DSA100, Krüss GmbH, Filderstadt, Germany) was used to evaluate the hydrophilicity of the membrane surface by measuring the static water contact angle on the membrane. A measure of 5 μL of water was dropped onto the membrane surface, and the contact angle of the water was measured. Each group of samples was tested at 10 different locations, and the average value was taken. The adhesion strengths of the ETFE and PTFE membranes were evaluated via a T-Peel test using a universal testing machine (34TM-5, Instron, Boston, MA, USA). The polyester fabric with a layer of adhesive (577H, GreatEastern Resins Industrial Co., Ltd., Dongguan, China) was used to bond the membranes. Prior to the tests, the specimen was dried at 60 °C for 12 h. The sample width was 25 mm and the peeling speed was 100 mm/min. At least five measurements were taken for each sample to measure the average.

## 3. Results and Discussion

### 3.1. ATR-FTIR and IR PiFM Analysis

**Figure 2:**
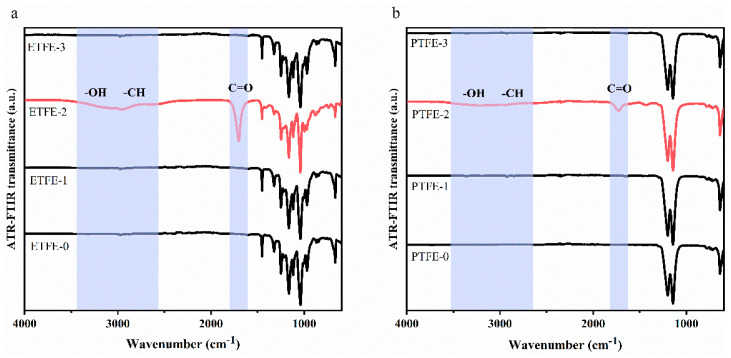
ATR-FTIR analysis of membrane before and after surface treatment: (**a**) ETFE membrane, (**b**) PTFE membrane.

ATR-FTIR was used to characterize the functional groups of ETFE and PTFE membranes before and after surface treatment. As shown in Figure 2, a series of strong absorption peaks of the untreated ETFE-0 membrane from 1350 to 1000 cm^−1^ were signed to the C–F bond of the CF_2_ group [13,14], and the sharp absorption peak at 1453 cm^−1^ was the C–H deformation vibrational bond [15,16]. For PTFE-0 membrane, peaks at 1203 cm^−1^ and 1147 cm^−1^ showed the stretching vibrational peak of CF_2_. The infrared spectrum of ETFE-1 membrane modified by He plasma was consistent with that of ETFE-0 membrane, and no new characteristic peaks were found in the PTFE-1 membrane, indicating that He plasma did not change the chemical compositions of the ETFE membrane.

**Figure 3:**
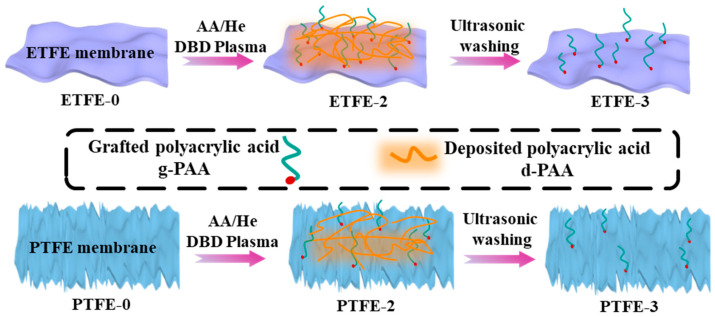
Mechanism of He/AA plasma modifications on ETFE and PTFE membranes.

Noticeably, the infrared spectrum of the ETFE-2 membrane sample modified by AA/He plasma showed a significant change compared to that of ETFE-0, with the appearance of a distinct C=O stretching vibration peak at 1705 cm^−^^1^ [11,15] and a broad peak in the range of 3700–2500 cm^−1^ containing OH stretching vibration peaks [17,18] and CH stretching vibration peaks [19]. These results indicate the appearance of some polyacrylic acid (PAA) on the surface of ETFE membranes after He/AA plasma treatment. The characteristic peaks of COOH were also observed on the surface of PTFE-2 membrane, but with weaker intensity compared to ETFE-2. However, these characteristic peaks of COOH were not observed in the infrared spectra of ETFE-3 and PTFE-3 membranes after ultrasonic washing with acetone. We hypothesize that PAA on the membrane surface contains both grafted polyacrylic acid (g-PAA) and deposited polyacrylic acid (d-PAA) after the He/AA plasma treatment, as shown in Figure 3. After ultrasonic washing with acetone, the d-PAA and a portion of the poorly grafted g-PAA were removed, and the trace amount of g-PAA remained on the surface did not reach the detection limit of ATR-FTIR. The ATR-FTIR signal is the accumulation of chemical information at the micron level depth of the surface, while the plasma modification may only occur at the nanometer level depth of the membrane surface. Thus, the signal of COOH peak cannot be well defined using ATR-FTIR.

**Figure 4:**
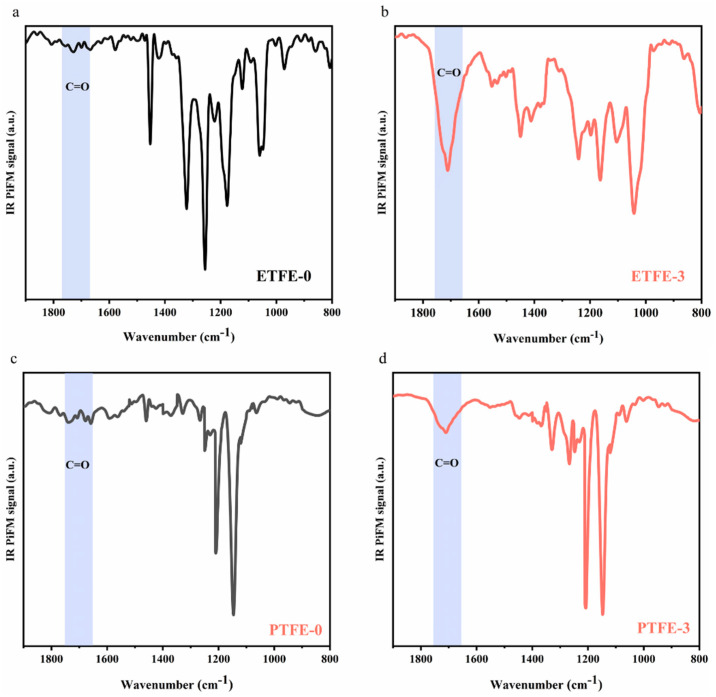
Comparison of IR PiFM spectra of membrane surface before and after treatment: (**a**) ETFE-0 membrane, (**b**) ETFE-3 membrane, (**c**) PTFE-0 membrane, and (**d**) PTFE-3 membrane.

To verify our hypothesis, IR PiFM was applied to analyze the surface chemistry of ETFE-0 and ETFE-3 membranes at the nano-layer level. The IR PiFM technique has been reported to resolve vibrational signals between a probing tip and material surface [20,21,22], obtaining infrared spectroscopy results with a resolution up to the nanometer level [23]. Taking ETFE-0 as an example, the nano-level infrared spectra of the membrane surface obtained by IR PiFM (Figure 4a) showed that a C–H deformation vibration peak at 1453 cm^−1^ and C–F peak at 950–1400 cm^−1^, which are consistent with the results obtained in ATR-FTIR (Figure 2a). Therefore, the IR PiFM spectra can precisely provide information on the chemical compositions of the sample surface.

In the ATR-FTIR spectrum, the C=O characteristic peak was not observed for the ETFE-3 membrane (Figure 2a), while the C=O characteristic peak at 1711 cm^−1^ could be clearly observed in the IR PiFM spectrum (Figure 4b), and the overall peak information was consistent with the ATR-FTIR characteristic peak observed for the ETFE-2 membrane. The IR PIFM analysis was also performed on the PTFE-0 membrane and the PTFE-3 membrane, and the C=O characteristic peak could also be found on the surface of the PTFE-3 membrane (Figure 4c,d). The test results of ETFE-3 membrane and PTFE-3 membrane by IR PiFM showed that ultrasonic washing with acetone can remove the d-PAA, but the surface still contains g-PAA at the nanoscale.

### 3.2. XPS Analysis

**Figure 5:**
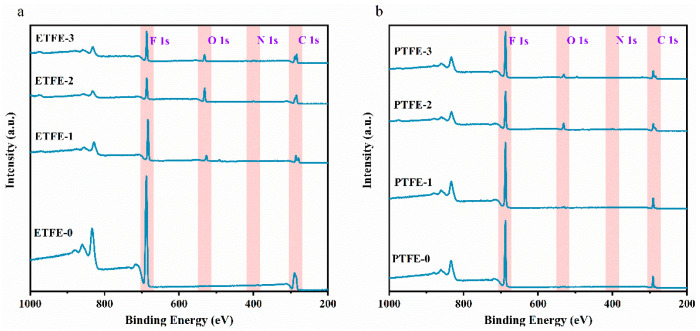
XPS spectra of membrane surface before and after treatment: (**a**) ETFE membrane and (**b**) PTFE membrane.

XPS was used to characterize the elemental concentration of the ETFE and PTFE membrane surfaces before and after plasma treatment. From Figure 5a and Table 2, it can be seen that the O element peak can be observed on the surface of the ETFE-1 membrane after helium plasma modification compared to the ETFE-0 membrane, the O element content can account for up to 8.52%, and the O/C can increase from 0.003 (ETFE-0 membrane) to 0.17, indicating that the He plasma modification will generate active sites on the membrane surface. These active sites, after being exposed to air, interact with the O_2_, H_2_O and CO_2_ molecules in air to form oxygen-containing groups [7,8].

In comparison with ETFE-0 membrane, the surface of ETFE-2 membrane modified by He/AA plasma has a strong peak of O element as the percentage of O element reached 20.3%. Moreover, the percentage ration of O/C increased from 0.003 (ETFE-0) to 0.36 (ETFE-2), and F/C decreased from 1.04 (ETFE-0) to 0.38 (ETFE-2). These results also confirm that the He/AA plasma treatment introduces a large amount of PAA into the membrane surface. The O element peak was still observed in the spectrum of ETFE-3 membrane after ultrasonic washing with acetone, and the percentage of O element content was up to 8.24%, accounting for the remaining g-PAA on the membrane surface. The F/C increased to 0.74 and O/C decreased to 0.15. This is because a large amount of d-PAA was removed after ultrasonic washing with acetone, thus making the F/C increase and O/C decrease.

As shown in Figure 5b and Table 2, the percentage of O element in the PTFE-1 membrane after He plasma treatment was only 1.54%. The O element peak on the surface of PTFE-2 and PTFE-3 membranes after He/AA plasma treatment became more evident. Additionally, the O element peak of PTFE-2 membrane was stronger than that of PTFE-3, indicating that there are two forms of PAA on the membrane surface, i.e., d-PAA and g-PAA. The change trends of F/C and O/C on the surface of PTFE membranes after plasma treatment are consistent with those of ETFE membranes.

**Figure 6:**
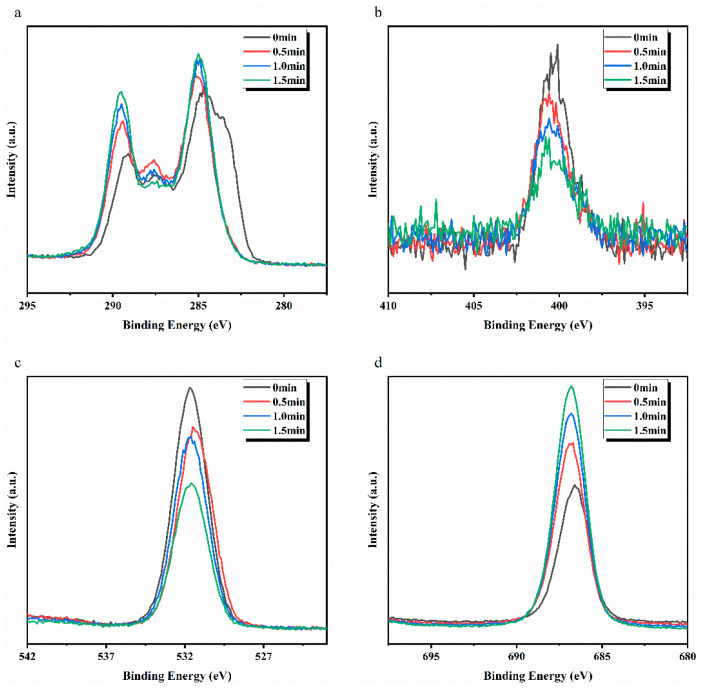
Comparison of peak intensity for each element on the surface of ETFE-2 membrane in a function of sputtering time: (**a**) C, (**b**) N, (**c**) O, and (**d**) F.

ETFE and PTFE membrane after plasma treatment were characterized using an Ar gas cluster-sputtering–XPS coupling technique to analyze the depth of the effect of plasma treatment on the surface modification effect of the membranes. XPS spectra were collected before the start of etching (*t* = 0 min) and after 0.5 min, 1.0 min, and 1.5 min of etching, respectively. As shown in Figure 6 and Table 3, the percentage of O elements on the surface of the ETFE-2 membrane was 20.3% before etching due to the presence of d-PAA and g-PAA, and the percentage of O elements was still as high as 13.92% after 1.5 min of etching, with O/C of 0.27 higher than 0.003 for ETFE-0 membrane and F/C of 0.68 smaller than 1.04 for ETFE-0 membrane. In addition, with the increase in the sputtering time, the sampling point is gradually close to the ETFE membrane bulk; thus, the C element peak gradually shifted to the high electron-binding energy, the O element intensity gradually decreased, and the F element peak intensity gradually increased. The weak signal of N element is mainly caused by impurities adsorbed on the surface of the membrane after being exposed to air. Similarly, the PTFE-2 membrane showed an increase in F/C and a decrease in the percentage of O element content and O/C after 1.5 min of etching.

**Figure 7:**
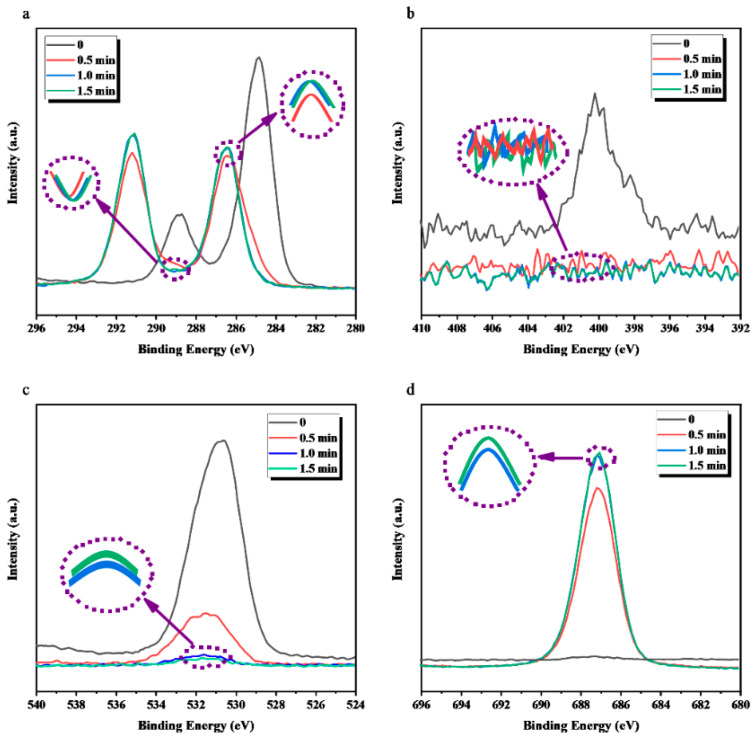
Comparison of peak intensity for each element on the surface of ETFE-3 membrane in a function of sputtering time: (**a**) C, (**b**) N, (**c**) O, and (**d**) F.

As shown in Figure 7, the change pattern of increasing sputtering time of each element peak intensity on the surface of the ETFE-3 membrane is the same as that of the ETFE-2 membrane. Different from the ETFE-2 membrane, the intensity of each element peak of the ETFE-3 membrane almost ceased to change after 1.0 min of etching, indicating the exposure of ETFE bulk at that time. This result is reasonable as only g-PAA is present on the surface of the ETFE-3 membrane after ultrasonic washing with acetone, and the thickness of PAA on ETFE-3 should be smaller than that on ETFE-2. Thus, the etching time at which the native characteristics of ETFE begin to appear would be shorter in the case of ETFE-3. 

### 3.3. Surface Morphlogy Analysis

**Figure 8:**
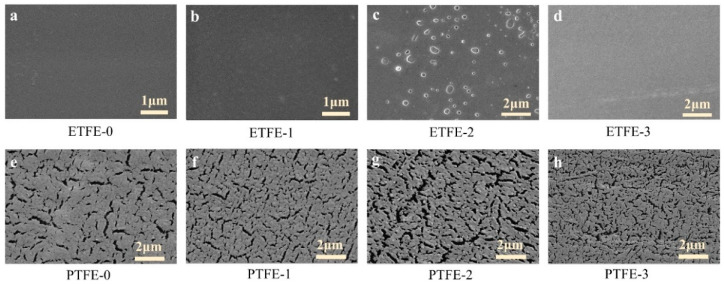
SEM images of membranes surface before and after plasma treatment: (**a**–**d**) ETFE membranes, and (**e**–**h**) PTFE membranes.

The surface morphology of ETFE and PTFE membranes before and after plasma treatment was characterized using SEM, as shown in Figure 8. The surface of the ETEF-0 membrane was dense and smooth (Figure 8a), and the ETFE-1 membrane became slightly rougher due to heat accumulation during the helium plasma treatment (Figure 8b). The surface of the ETFE-2 membrane showed some aggregates, possibly due to the presence of g-PAA and d-PAA (Figure 8c). No further PAA aggregates were observed on the surface of the ETFE-3 membrane after acetone ultrasonic washing because the acetone washing removed the d-PAA from the membrane surface (Figure 8d). The surface of the PTFE-0 membrane has a mass of irregular fine cracks, which were produced during the cutting process of the PTFE membrane (Figure 8e). The surface of the PTFE-1 membrane after He plasma treatment was still dominated by fine cracks (Figure 8f). The presence of PAA aggregates observed on the surface of the ETFE-2 and PTFE-2 membranes also corroborates with the COOH characteristic peak observed in ATR-FTIR results. As with the ETFE-3 membrane, no obvious PAA aggregates were observed in the case of the PTFE-3 membrane after ultrasonic washing with acetone.

**Figure 9:**
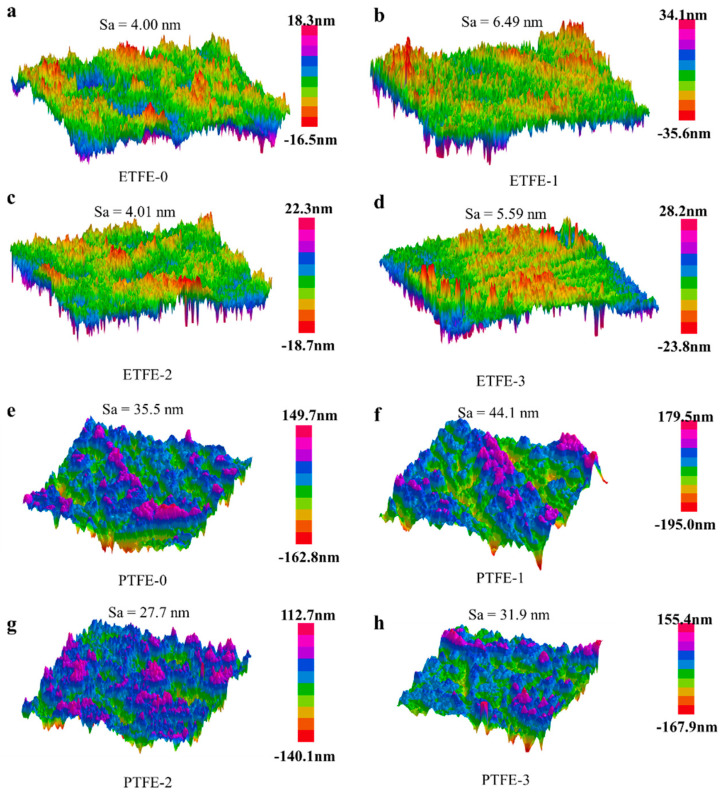
AFM imaging of membranes before and after surface treatment: (**a**–**d**) ETFE membranes and (**e**–**h**) PTFE membranes. Sa is average surface roughness of the scanned area.

The surface morphologies of the ETFE and PTFE membranes before and after plasma treatment were tracked using AFM, as shown in Figure 9. The surface of the original ETFE membrane was relatively flat with an average surface roughness (Sa) value of 4.00 nm (Figure 9a). The Sa value of the ETFE-1 membrane after He plasma treatment increased to 6.49 nm (Figure 9b), indicating that the etching effect of He plasma on the surface of the ETFE membrane increased the surface roughness of the membrane. The Sa value of the ETFE-3 membrane after He/AA plasma treatment declined to 4.01 nm, possibly due to the uniform distribution of the PAA layer on the surface of the membrane (Figure 9c). The Sa value of the ETFE-3 membrane after acetone ultrasonic washing changed to 5.59 nm because the acetone ultrasonic washing removed the uniformly distributed d-PAA from the membrane surface, and only the randomly distributed g-PAA remained on the surface. Thus, the surface roughness of the ETFE-3 membrane after washing was greater than that of the ETFE-2 membrane (Figure 9d).

The PTFE-0 membrane had a fine-textured surface (Figure 9e) with a Sa value of 35.5 nm, much larger than that of the ETFE-0 membrane with a Sa value of 4.00 nm. After He plasma treatment, the surface roughness increased as well as the ETFE membrane with a Sa value of 44.1 nm (Figure 9f). However, the Sa value of the PTFE-2 membrane after He/AA plasma treatment was 27.7 nm (Figure 9g), and the Sa value of the PTFE-3 membrane after ultrasonic washing with acetone was 31.9 nm (Figure 9h), both of which were smaller than the Sa value of 35.5 nm for the PTFE-0 membrane. This is because the deposited PAA layer on the surface of the PTFE membrane after He/AA plasma treatment can reduce the roughness of the original PTFE membrane to a certain extent. After the removal of d-PAA by acetone ultrasonic washing, the residual g-PAA on the membrane surface has a certain effect on filling the original fine lines on the surface of the original PTFE membrane, which leads to the Sa value of the PTFE-3 membrane still being smaller than that of the original PTFE, which also to a certain extent reflect the nanometer scale of g-PAA produced by the He/AA plasma treatment on the membrane surface.

### 3.4. Hydrophilicity Analysis

**Figure 10:**
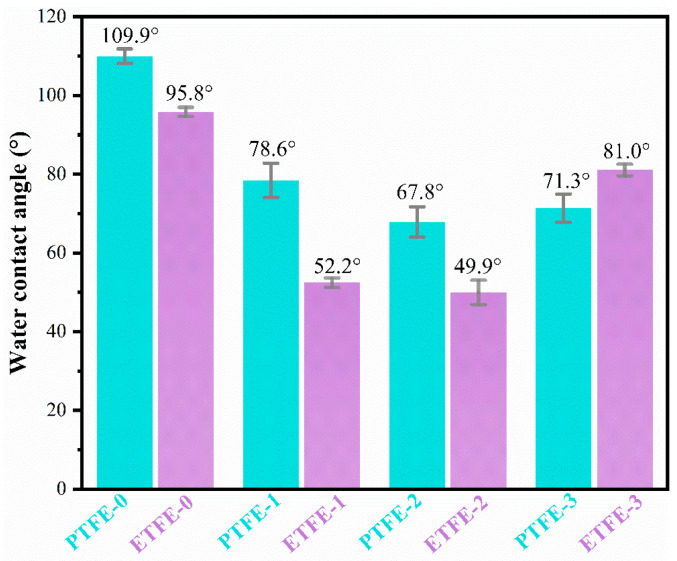
Comparison of the water contact angle of membrane before and after surface treatment.

The change in hydrophilicity of the fluorine resin membrane surface before and after plasma treatment was analyzed by the water contact angle test. As shown in Figure 10, the water contact angle of the plasma-treated ETFE membrane and plasma-treated PTFE membrane decreased compared to their untreated membrane.

The water contact angle of the ETFE-1 and PTFE-1 membranes treated with helium plasma decreased significantly compared to their untreated membranes. Specifically, the ETFE-1 membrane decreased from 95.83° to 52.28°, a decrease of 45.45% relative to the ETFE-0 membrane. The PTFE-1 membrane decreased from 109.9° to 78.7° before treatment, which is a 28.39% decrease relative to the PTFE-0 membrane. Not only is the roughness of the membrane surface increased after helium plasma treatment, but some reactive groups are also generated on the membrane surface, which increases the polarity and surface energy; thus, the hydrophilicity of the membrane surface is significantly improved.

The surface water contact angle of the ETFE-2 membrane modified by He/AA plasma was 49.98°, a decrease of 47.84% relative to the ETFE-0 membrane. The contact angle of the PTFE-2 membrane decreased from 109.9° in the PTFE-0 membrane to 67.8°, a decrease of 38.30% relative to the ETFE-0 membrane. The contact angle was further reduced and the hydrophilicity of the membrane was enhanced due to the introduction of PAA. The ETFE-3 and PTFE-3 membranes showed an increase in the water contact angle after ultrasonic washing with acetone compared to before washing, increasing to 81.0° for the ETFE-3 membrane and 71.3° for the PTFE-3 membrane, which was due to the removal of d-PAA, resulting in a decrease in the PAA content on the membrane surface; thus, the hydrophilicity decreased and the water contact angle increased. Compared with the smooth surface of the ETFE membrane, the surface of the PTFE membrane is rougher and the PAA in the surface crevices is more difficult to be removed during acetone sonication. As a result, the water contact angle of the PTFE-3 membrane is smaller than that of the ETFE-3 membrane.

### 3.5. Adhesion Analysis

**Figure 11:**
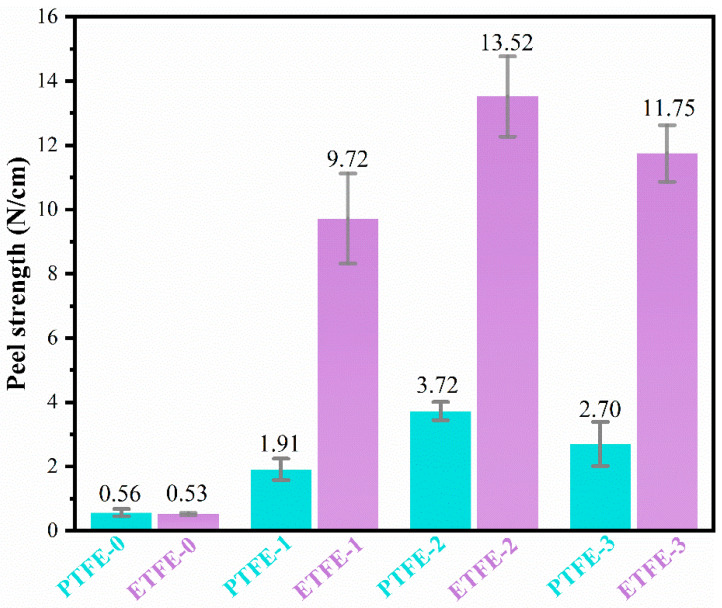
Comparison of the peel strength of ETFE and PTFE membranes before and after surface treatment.

The surface adhesion strength of fluororesin membranes before and after plasma treatment was assessed by a T-peel test. As shown in Figure 11, the peel strength of the ETFE and PTFE membranes after plasma treatment was significantly improved in comparison with the pristine membranes. As we mentioned above, the He plasma treatment may result in the improved surface roughness and hydrophilicity of the membranes, thus increasing the peel strength of the PTFE-1 membrane and ETFE-1 membrane compared to the respective untreated membranes. The He/AA plasma treatment is able to introduce a great amount of PAA on the membrane surface. The peel strength of the PTFE-2 membrane was further increased from 1.91 N/cm (PTFE-1) to 3.72 N/cm, and the peel strength of the ETFE-2 membrane was increased from 9.72 N/cm (ETFE-1) to 13.52 N/cm. The polar PAA has good compatibility with the adhesive, which increases the interfacial interactions between the adhesive and the membranes and improves the bond strength of the membranes. After ultrasonic washing with acetone, the removal of d-PAA weakened the interaction between the adherent and membranes, causing a slight decrease in the peel strength. In comparison, the peel strength of ETFE was much higher than that of PTFE under all treatment conditions, demonstrating that the perfluorinated resin is more inert and more difficult to modify than the non-perfluorinated resin. 

## 4. Conclusions

In this research, atmospheric pressure DBD plasma was used to modify the surface properties of the ETFE and PTFE membranes under the He and mixed He/AA atmospheres. The effects of atmospheres on the surface modification were investigated, and the related mechanisms were analyzed. ATR-FTIR and IR PiFM tests showed that the He plasma treatment did not cause structural damage to the membrane, and the AA/He plasma treatment caused the membrane to have a stable nano-level g-PAA presence, and the surface amount of PAA on ETFE surface is more than PTFE. XPS analysis showed that the He plasma treatment produced active sites on the membrane surface, which led to the ratio of F/C on the membrane surface decreasing slightly. On the other hand, the He/AA plasma introduced PAA to the membrane surface, which decreased the ratio of F/C on the membrane surface significantly. Morphology analysis by SEM and AFM showed that He plasma treatment produced only nanoscale etching on the membrane surface. In comparison, the He/AA plasma treatment resulted in PAA clusters on the membrane surface. The active sites and PAA on the membrane surface after plasma treatment resulted in the improved hydrophilicity and adhesion strength of the membrane.

To assess the effects of plasma modification on the bondability of fluororesine membranes, T-peel tests were conducted to measure the peel strength of the membrane surface. The surface peel strength of the ETFE and PTFE membranes noticeably improved after He plasma and He/AA plasma treatments, and the degree of improvement was more pronounced for ETFE membranes with non-perfluorinated structures. With the He/AA plasma treatment, the peel strength of the ETFE membrane was increased from 0.53 N/cm to 13.52 N/cm, and the peel strength of the PTFE membrane was increased from 0.56 N/cm to 3.72 N/cm. These results demonstrate that the surface modification by plasma could provide more opportunities for the practical applications of fluorine resin membranes in the fabrication of protective clothing. Moreover, the atmospheric pressure DBD plasma modification method is facile as it can modify the membrane surface in one step, and the nano-scaled modification effect under He/AA atmosphere was stable, displaying great potential in membrane processing and engineering in a practical setting.

## Figures and Tables

**Table 1:** Experimental conditions and specific sample numbers.

	Untreated	He Plasma Treatment	He/AA Plasma Treatment	He/AA Plasma Treatment + Acetone Ultrasonic
ETFE	ETFE-0	ETFE-1	ETFE-2	ETFE-3
PTFE	PTFE-0	PTFE-1	PTFE-2	PTFE-3

High-voltage AC power supply working voltage is 35 V; gas flow rate is 200 mL/min; plasma processing time is 15 s; experimental environment temperature is 25 °C.

**Table 2:** Percentage of elemental content of ETFE and PTFE membranes before and after surface treatment.

	C (%)	N (%)	O (%)	F (%)	F/C	O/C
ETFE-0	48.83	0.04	0.17	50.96	1.0436	0.0035
ETFE-1	49.31	0.16	8.52	42.01	0.8519	0.1727
ETFE-2	56.00	2.17	20.3	21.54	0.3846	0.3625
ETFE-3	52.51	0.09	8.24	39.16	0.7457	0.1569
PTFE-0	35.65	0.02	0.54	63.79	1.7893	0.0151
PTFE-1	35.78	0.22	1.47	62.54	1.7479	0.0410
PTFE-2	41.73	2.25	10.72	45.31	1.0857	0.2568
PTFE-3	40.57	0.71	5.79	52.93	1.3046	0.1427

**Table 3:** Analysis of elemental content of ETFE-2 and PTFE-2 membranes as a function of sputtering time.

Sputtering Time	C (%)	N (%)	O (%)	F (%)	F/C	O/C
ETFE-2 0 min	56.00	2.17	20.29	21.54	0.3846	0.3625
ETFE-2 0.5 min	49.72	1.59	22.34	26.35	0.5299	0.4493
ETFE-2 1.0 min	50.43	1.34	17.91	30.32	0.6012	0.3551
ETFE-2 1.5 min	50.51	1.00	13.92	34.57	0.6844	0.2755
PTFE-2 0 min	41.73	2.25	10.72	45.31	1.0857	0.2568
PTFE-2 1.5 min	40.24	1.12	7.93	50.71	1.2601	0.1970

## Data Availability

Not applicable.
